# Multi-organ dysfunction across the neonatal encephalopathy spectrum

**DOI:** 10.1038/s41390-025-03978-2

**Published:** 2025-03-19

**Authors:** Lynn Bitar, Rachel L. Leon, Yu-Lun Liu, Srinivas Kota, Lina F. Chalak

**Affiliations:** 1 Division of Neonatal-Perinatal Medicine, Department of Pediatrics, University of Texas Southwestern Medical Center, Dallas, TX USA; 2 Peter O’Donnell Jr. School of Public Health University of Texas Southwestern Medical Center, Dallas, TX USA

## Abstract

**Background:**

Neonatal hypoxic-ischemic encephalopathy (HIE), the leading cause of neonatal encephalopathy (NE), primarily affects the central nervous system and is associated with multi-organ dysfunction (MOD) and long-term complications. Research often focuses on moderate to severe NE, with limited data on mild cases.

**Objective:**

To investigate the incidence and severity of MOD in neonates with mild NE and explore its association with HIE severity.

**Methods:**

Term neonates with NE related to HIE diagnosis between 2009 and 2023 were included. Sarnat staging was used to classify cases into mild and moderate/severe. MOD was assessed on days 1 and 3 post-birth through echocardiography, troponin levels, creatinine levels, urine output, and liver function tests.

**Results:**

Among 452 neonates with HIE (185 mild, 267 moderate/severe), 57% had liver injury, 55% cardiac injury, and 44% kidney injury in the first day of life. Neonates with mild NE had a MOD rate of 23%, lower than the 37% observed in moderate/severe (*p* = 0.002). When compared to mild, infants with moderate/severe NE had significantly higher incidences of cardiac (69% vs. 31%; *p* < 0.001), renal (49% vs. 38%; *p* = 0.067), and hepatic abnormalities (65% vs. 45%; *p* = 0.005).

**Conclusions:**

This study highlights the risk of extra-cranial organ injury even in infants with mild NE, stressing the importance of monitoring all regardless of severity.

**Impact:**

- Comprehensive study prospectively evaluating end-organ dysfunction in a cohort of neonates diagnosed with mild, moderate, and severe NE.

## Introduction

Perinatal asphyxia (PA) is a complex, multi-organ condition resulting from compromised blood flow in the peripartum period, leading to decreased oxygen supply and tissue ischemia,^1–4^ affecting 1–6 per 1000 live births.^4–7^ Hypoxic-ischemic encephalopathy (HIE) is the most common form of neonatal encephalopathy (NE), and is diagnosed when perinatal asphyxia affects the neurologic status of neonates.^8–10^ Although more focus is directed towards its impact on the brain, perinatal asphyxia is a systemic condition that affects multiple organ systems.^11,12^ To protect vital organs such as the brain, heart, and adrenals during oxygen deprivation, neonatal hypoxia triggers the diving reflex, redirecting blood away from organs such as the liver and kidneys.^13^ Importantly, the severity of multiorgan dysfunction (MOD) is not always correlated with the severity of encephalopathy, indicating that significant MOD can occur across the whole HIE spectrum.^5,14–16^

Differentiating HIE from other causes of NE requires a comprehensive clinical evaluation: The history of a perinatal event, such as abnormal fetal heart tracings, uterine rupture, or cord prolapse, is critical in suspecting HIE. Apgar scores of less than 5 at 1, 5, and 10 min, along with the need for extensive resuscitation, are common in HIE. Additionally, blood gas analysis identifies severe metabolic acidosis (pH < 7.0 and base deficit >12 mmol/L), reflective of hypoxia and ischemia.^17–19^ The severity of NE can be clinically classified using the modified Sarnat score as mild (stage 1), moderate (stage 2), and severe (stage 3).^18,20^ Additionally, the extent of the brain injury can be assessed using electroencephalogram (EEG) and neuroimaging, with brain magnetic resonance imaging (MRI).^20–22^

Previous studies on cooled neonates with moderate and severe encephalopathy have described MOD, including cardiac,^23–25^ renal,^26^ and hepatic involvement.^27^ These studies have identified several diagnostic tools for detecting MOD in HIE. However, the extent and severity of multiorgan involvement in mild NE remain unclear. To address this, we evaluated the extent of MOD in a cohort of neonates with HIE. Our objectives were twofold: (1) to describe the incidence of cardiac, renal, and hepatic dysfunction in neonates with mild NE compared to those with moderate/severe, and (2) to investigate the association between the severity of NE and the extent of MOD.

## Methods

### Study design and population

This single-center retrospective cohort study included newborns with NE admitted to the Neonatal Intensive Care Unit (NICU) at Parkland Hospital, between January 2009 and July 2023, one of the largest inborn delivery services in the United States, with approximately 12,000 deliveries annually. NE was defined as evidence of encephalopathy within the first six hours of life, identified through clinical examination using a modified Sarnat exam, without requiring immediate evidence of hypoxia-ischemia.^9^ HIE was subsequently diagnosed in neonates with clinical encephalopathy if additional criteria indicating hypoxia-ischemia were met, including: (1) abnormal cord blood gas (pH < 7.0 or base deficit ≥ 12 mmol/L), (2) sentinel events (e.g., uterine rupture, placental abruption, or cord prolapse), and (3) MRI findings consistent with ischemic brain injury (e.g., basal ganglia or watershed pattern injury).^9,28^

We excluded preterm newborns ( < 35 weeks gestation), those with congenital anomalies, and those with additional neurologic disorders such as congenital brain malformation, infections affecting the brain, interventricular hemorrhage (IVH), periventricular leukomalacia (PVL), perinatal strokes, etc… Through retrospective review, we collected maternal demographic and pregnancy-related characteristics including age, comorbidities, gravidity, parity, prenatal care, and perinatal complications. We also gathered neonatal data including universal cord blood gas screening for fetal acidosis, Apgar scores at 1, 5, and 10 min, organ specific complications, and length of hospital stay.

**Fig. 1:**
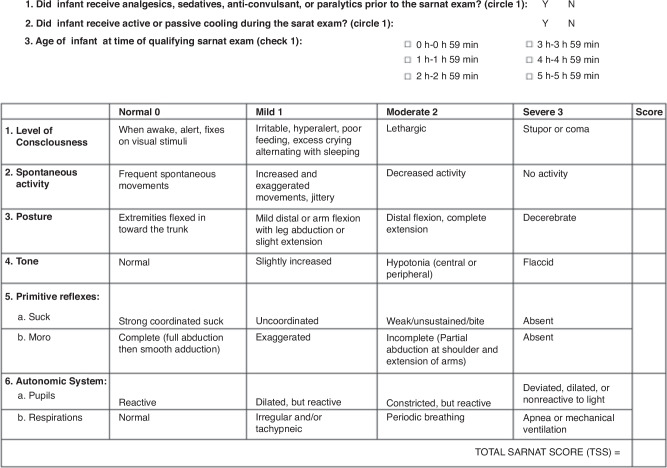
Sarnat scoring system for assessing neonatal encephalopathy.

Institutional protocol for HIE neonates provides guidelines for evaluation of all neonates for cardiac, renal, and hepatic injury in the first days after birth. We also conducted an electronic medical record review for all neonates to address concerns regarding other organ involvement. All newborns were evaluated per protocol between 1 and 6 h after birth using a modified Sarnat exam by trained clinicians to determine the severity of encephalopathy that included (1) level of consciousness, (2) spontaneous activity, (3) posture, (4) tone, (5) primitive reflexes (suck, moro), and (6) autonomic system (pupils, heart rate, respirations), with scores of normal (0), mild (1), moderate (2), or severe (3) (Fig. 1).^20,29^ The Total Sarnat Score (TSS) was determined by summing the scores for each of the six categories, which ranges from 0 to 18, where 0 represents normal in all six categories, and 18 represents severe encephalopathy in all six categories.^18,29^ The TSS scores were calculated; The worst score in the first six hours was used for decision making about cooling. EEG and brain MRI were performed for all patients with NE in accordance with our institutional protocol. MRI abnormalities included diffuse white matter and basal ganglia involvement and/or cortical changes consistent with an ischemic injury.

In this study, neonates were classified into two groups based on the severity of encephalopathy: moderate/severe encephalopathy with three or more abnormal (score 2 or 3) categories, and mild encephalopathy with one to two abnormal categories (Fig. 1).

For all newborns with moderate and severe encephalopathy, whole-body therapeutic hypothermia (TH) was initiated within 6 h of life (average of 4 h of life) as per the National Institute of Child Health and Human Development (NICHD) guidelines.^4,19^ A servo-controlled blanket (Blanketrol II, Cincinnati Sub-Zero Products LLC, OH) was utilized to maintain a core body temperature of 33.5 °C for 72 h, after which rewarming was conducted at a rate of 0.5 °C per one to two hours over the next six hours.^30^ Newborns with mild encephalopathy received normothermia as standard care. TH was initiated according to the NICHD late hypothermia protocol for newborns with mild NE who progressed to more severe encephalopathy or developed seizures beyond six hours but within the first 24 hs of life.^31^

### Organ injury evaluation

Investigation of MOD in the heart, liver and kidney in neonates with HIE, was based on a review of relevant literature of organs most commonly affected in HIE.^16,32–38^ These measures included using serial imaging and blood biomarkers. In the first 24 h of life (12 ± 6 h), and on day 3 after birth, cardiac function was assessed with Troponin T levels followed by echocardiography when clinically indicated. Renal function was assessed with creatinine levels and urine output. Liver function was assessed with serial aspartate aminotransferase (AST) and alanine transaminase (ALT) levels on day 1 and 3 of life. Cardiac injury was defined as Troponin T > 0.1 ng/mL or any evidence of cardiac injury on echocardiography (myocardial dysfunction, wall motion abnormalities, pulmonary hypertension).^33,34,39,40^ Renal injury was defined by creatinine level > 1 mg/dL in the presence of oliguria ( < 1 mL/kg/h).^33,34^ Hepatic injury was defined by AST or ALT > 100 U/L.^16,32–35,41^

### Statistical analysis

For patient characteristics and rate of MOD, we reported descriptive statistics including median with interquartile range [IQR], mean with standard deviation (SD), and frequency with percentage. Depending on the normality of continuous variables, we compared groups with the unpaired t-test or the non-parametric Wilcoxon rank-sum test. The Shapiro-Wilk test was used to assess the normality assumption. For categorical variables, we used ${\chi }^{2}$ or Fisher’s exact test. To determine associations between Troponin T, Creatinine, ALT, AST and the total Sarnat score we used non-parametric Spearman’s correlation coefficients. Univariate logistic regression was performed to evaluate the associations between multi-organ injury and the severity of encephalopathy, as well as the effect of Troponin T level on mortality, with odds ratios and 95% confidence intervals reported. Statistical significance was defined as a two-sided *p* value less than 0.05. All statistical analyses were conducted with R version 4.3.1 (R Foundation for Statistical Computing, Vienna, Austria).

### Study approval

This study was approved by the Institutional Review Board of the University of Texas Southwestern Medical Center, Dallas, USA.

## Results

### Study population

**Fig. 2:**
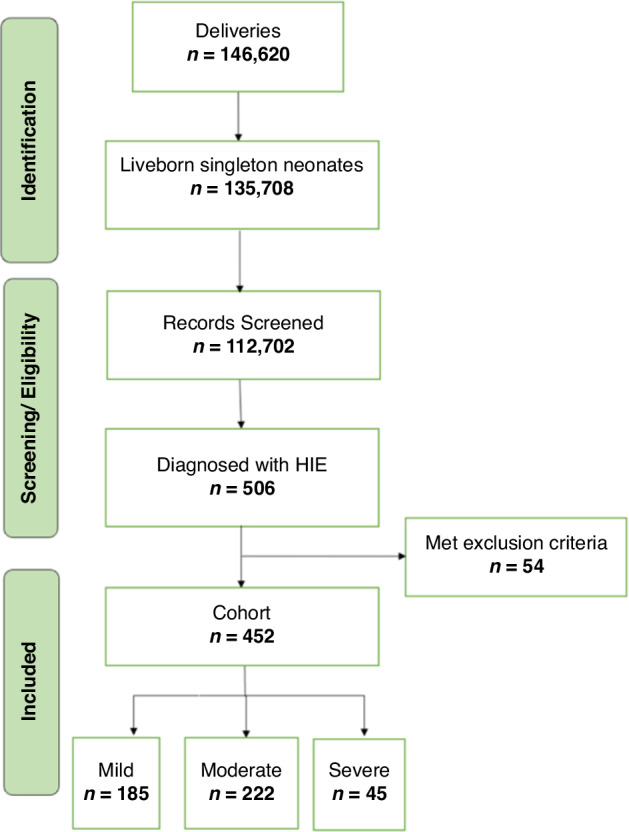
Flow diagram depicting the cohort distribution from January 2009 to July 2023.

Between January 2009 and July 2023, a total of 146,620 newborns were delivered at Parkland Hospital (Fig. 2). Of these, 135,708 were liveborn singleton neonates, of which 112,702 had blood gas available, and 3870 experienced metabolic acidosis or perinatal asphyxia. Ultimately, 506 neonates had perinatal acidosis and acute events consistent with attributing NE cause to HIE. After excluding preterm newborns ( < 35 weeks of gestation) and those with other neurological conditions at birth (congenital brain malformation, infections affecting the brain, IVH, PVL), the final cohort contained 452 patients with Group 1 including 185 newborns with mild NE (41%) who received supportive care, and Group 2 including 267 newborns with moderate to severe NE (59%) who underwent therapeutic hypothermia.

### Demographic, obstetric, and perinatal characteristics

Demographic, obstetric, and perinatal characteristics are presented in Table 1. Maternal age, parity, and gravidity were similar between groups. The cohort was predominantly White (66%), with 22% Black, and 69% identifying as Hispanic. Cesarean deliveries accounted for 70% of the cases, and 26% had chorioamnionitis. When comparing mild to moderate/severe neonates with NE, there were no significant differences in obstetrical gestational age (39 vs. 39 weeks respectively, *p* = 0.689), birth weight (3226 vs. 3250 grams respectively, *p* = 0.921), and head circumference (34 vs. 34 cm respectively, *p* = 0.296). However, significant differences were noted in Apgar scores at 1 min (3.0 [2.0, 4.0] vs. 2.0 [1.0, 3.0] respectively, *p* < 0.001), 5 min (6.0 [4.0, 8.0] vs. 5.0 [3.0, 7.0] respectively, *p* < 0.001), and 10 min (7.0 [5.0, 8.0] vs. 6.0 [4.0, 7.0] respectively, *p* < 0.001). Overall, 28% of neonates had seizures during the hospital stay. In neonates with moderate to severe NE, there was a significantly longer hospital stay compared to mild NE (14 [10,26] vs. 7 [5,12] days, *p* < 0.001). The overall survival rate was 93%, with 98% for mild cases and 90% for moderate to severe cases (*p* < 0.001).

**Table 1:** Baseline maternal and neonatal characteristics of the cohort, stratified by HIE severity.

Maternal Characteristics	All (*n* = 452)	Mild (*n* = 185)	Moderate/Severe (*n* = 267)	*P* value
**Maternal age (years)**	27 [22,33]	27 [23,34]	28 [22,33]	0.81
**Gravidity**	2 [1,4]	2 [1,4]	2 [1,3]	0.73
**Parity**	1 [0, 2]	1 [0, 2]	1 [0, 2]	0.55
**Route of Delivery,** ***N*. (%)**				
Vaginal	135/450 (30)	59 (32)	76/265 (29)	0.53
C-section	315/450 (70)	126 (68)	189/265 (71)	
**Prenatal care,** ***N*. (%)**	430/446 (96)	180 (97)	250/261 (96)	0.55
**Pre-eclampsia,** ***N*. (%)**	131/450 (29)	49 (27)	82/265 (31)	0.36
**Gestational Diabetes,** ***N*. (%)**	62/450 (14)	29 (16)	33/265 (13)	0.40
**Chorioamnionitis,** ***N*. (%)**	108/417 (26)	51 (28)	57/232 (25)	0.56
**Neonatal Characteristics**	**All (*n*** = **452)**	**Mild (*n*** = **185)**	**Moderate/Severe (*n*** = **267)**	***P*** **value**
**Race,** ***N*. (%)**				
White	295/404 (73)	133 (74)	162/225 (72)	0.83
Black	96/404 (24)	40 (22)	56/225 (25)	
Asian	13/404 (3)	6 (3)	7/225 (3)	
**Ethnicity,** ***N*. (%)**				
Non-Hispanic	122/394 (31)	53/176 (30)	69/218 (32)	0.82
Hispanic	272/394 (69)	123/176 (70)	149/218 (68)	
**Sex,** ***N*. (%)**				
Male	252/450 (56)	110 (59)	142/265 (54)	0.25
Female	198/450 (44)	75 (41)	123/265 (46)	
**Estimated obstetrical gestational age**	39 [38,40]	39 [38,40]	39 [38,40]	0.68
**Apgar 1** **min**	2.0 [1.0, 4.0]	3.0 [2.0, 4.0]	2.0 [1.0, 3.0]	<0.001*
**Apgar 5** **min**	6.0 [3.0, 7.0]	6.0 [4.0, 8.0]	5.0 [3.0, 7.0]	<0.001*
**Apgar 10** **min**	6.0 [4.0, 8.0]	7.0 [5.0, 8.0]	6.0 [4.0, 7.0]	<0.001*
**Head circumference**	34 [33,35]	34 [33,35]	34 [33,35]	0.29
**Birth weight**	3235 [2860,3638]	3226 [2885,3680]	3250 [2830,3600]	0.92
**Seizures on EEG during hospital stay, N. (%)**	72/262 (27)	14/55 (25)	58/207 (28)	0.83
**Neonatal sepsis,** ***N*. (%)**	15/450 (3)	4 (2)	11/265 (4)	0.3
**Total hospital days**	12 [7,22]	7 [5,12]	14 [10,26]	<0.001*
**Death,** ***N*. (%)**	30 (7)	3 (2)	27 (10)	<0.001*

When the normality assumption was violated, median and interquartile range (IQR) were reported, and the Wilcoxon rank sum test was used to assess statistical significance.

*Indicates statistical significance (*p* < 0.05).

### Radiologic and organ specific biomarkers among all HIE neonates

Neonates with moderate to severe NE had a significantly higher rate of MRI abnormalities compared to those with mild NE (69% vs. 33%, *p* = 0.04). Among the whole cohort, 57% of neonates had laboratory evidence of liver injury, 55% had cardiac injury, and 44% had kidney injury. Neonates with moderate and severe NE showed a significantly higher incidence of liver (65% and 64% vs. 45% respectively; *p* = 0.013), cardiac (70% and 59 vs. 31% respectively; *p* < 0.001), and kidney (50% and 41% vs. 38% respectively; *p* = 0.090) injury compared to those with mild NE (Table 2).

**Table 2:** Distribution of organ injuries stratified by HIE grade.

	All (*n* = 452)	Mild (*n* = 185)	Moderate (*n* = 222)	Severe (*n* = 45)	*P* value
**Total number of organs injured, No. (%)**					<0.001*
**0**	168 (37)	103 (56)	52 (23)	13 (29)	
**1**	144 (32)	40 (22)	85 (38)	19 (42)	
**2**	92 (20)	30 (16)	55 (25)	7 (15)	
**3**	48 (11)	12 (6)	30 (13)	6 (13)	
**Liver injury, No. (%)**	129/228 (57)	44/97 (45)	69/106 (65)	16/25 (64)	0.013*
**Heart injury, No. (%)**	201/367 (55)	43/137 (31)	136/193 (70)	22/37 (59)	<0.001*
**Kidney injury, No. (%)**	142/321 (44)	49/130 (38)	80/159 (50)	13/32 (41)	0.09

Cutoff values AST > 100 U/L; ALT > 100 U/L; Creatinine > 1 mg/dL + oliguria; Troponin T > 0.1 ng/mL or abnormal echo findings.

Per standard protocol, organ-specific biomarkers were recorded on days 1 and 3 after birth for all neonates with HIE (Table 3). AST levels were significantly higher in moderate/severe compared to mild NE (130.0 [78.5, 258.0] vs. 97.0 [72.0, 191.5], *p* = 0.047). Similarly, Troponin T levels were also higher in moderate/severe NE compared to mild NE (0.23 [0.15, 0.45] vs. 0.10 [0.06, 0.19], *p* < 0.001).

**Table 3:** Day 1 Laboratory biomarker levels.

Variable	All (*n* = 452)	Mild (*n* = 185)	Moderate/Severe (*n* = 267)	*P* value
**AST**	116.5 [75.0, 239.5]	97.0 [72.0, 191.5]	130.0 [78.5, 258.0]	0.04*
**Creatinine**	0.94 [0.77, 1.12]	0.92 [0.77, 1.09]	0.97 [0.78, 1.12]	0.45
**Troponin T**	0.19 [0.12, 0.38]	0.10 [0.06, 0.19]	0.23 [0.15, 0.45]	<0.001*

When evaluating the direction of change in organ-specific biomarkers from day 1 to day 3, there was an improvement in AST, creatinine, and troponin T levels in all HIE groups (*p* < 0.05).

### Association between multi-organ injury and neurological encephalopathy severity

Univariate logistic regression demonstrated a significant association between the severity of encephalopathy and the likelihood of organ injury in neonates. Specifically, as the degree of neurological encephalopathy worsens, the odds of having at least one organ injury more than doubles (OR = 2.8 [95% CI: 1.91, 4.08], *p* < 0.001). Similarly, as the degree of encephalopathy worsens, the odds ratio of injuries to two organs is 2.2 times higher (OR = 2.2 [95% CI: 1.4, 3.3], *p* < 0.001) and to three organs is 2.9 times higher (OR = 2.9 [95% CI: 1.7; 4.9], *p* < 0.001). An association was also found between increased Troponin T levels and a higher mortality (OR = 1.86 [95% CI: 1.32, 2.63], *p* < 0.001).

The association between the severity of HIE and liver dysfunction (AST *r* = 0.032, ALT *r* = 0.089 *p* = 0.022) was also noted. Specifically, compared to neonates with mild NE, those with moderate NE were seven times more likely to die (OR = 7.0 [95% CI: 2.1, 23.7], *p* = 0.002), while those with severe NE were nearly six times more likely to die (OR = 5.9 [95% CI: 1.3, 27.5], *p* = 0.002). On binomial logistic regression analysis, a higher degree of encephalopathy was also associated with an increased mortality rate.

## Discussion

This study addresses a gap in understanding MOD across the complete spectrum of NE severity, particularly emphasizing MOD surveillance in mild NE for counseling and management. When laboratory testing is systematically performed to assess for end-organ damage, we demonstrate that MOD affects 1 out of 4 of neonates with mild NE. These results highlight that all neonates with NE attributable to asphyxia, regardless of severity, should be screened and monitored for end-organ damage.

### Cardiac injury

Systemic hypoxia and subsequent cardiac output redistribution in HIE can lead to reperfusion injury affecting the heart.^42^ Using a Troponin T cut-off of 0.1 ng/ml along with abnormal findings on echocardiography to indicate cardiac injury, our study showed a 69% incidence of cardiac injury in neonates with moderate to severe NE, consistent with prior reports.^24,33,35,39,43–47^ In line with prior studies reporting an incidence of cardiac injury between 50 and 78% in neonates with moderate to severe NE, our cohort showed a concordant rate of cardiac injury of 69%. Surprisingly, 31% of the mild NE group also had cardiac injury. Troponin T has been recognized as an early predictor of mortality in neonates with moderate to severe NE. In line with Boo et al.‘s study, we found an association between elevated Troponin T levels and higher mortality (OR = 1.86 [95% CI: 1.32, 2.63], *p* < 0.001).^48^ Notably, all neonates who died from HIE had elevated Troponin T, including three with initially mild encephalopathy that progressed. This highlights the importance of close cardiac monitoring.

### Renal injury

HIE can reduce blood flow to the kidneys, leading to ischemic damage and acute kidney injury (AKI).^49^ Prior studies report renal dysfunction rates between 22% and 70% in moderate to severe NE cases.^27,41–43^ In our study, approximately 44% of neonates with NE experienced renal injury. The kidney is considered the most commonly affected organ in HIE due to its vulnerability to hypoperfusion from high intrauterine vascular resistance, elevated plasma renin, and low glomerular filtration.^41,50^ Interestingly, the mild group in our cohort showed a high rate of renal dysfunction (38%), indicating that the kidneys are commonly affected even in mild cases. Long-term implications of elevated creatinine in moderate to severe cases include the potential for renal replacement therapy, with a minority requiring renal transplant.^39,51,52^

### Hepatic injury

Liver injury in HIE primarily results from hypoperfusion due to compromised blood flow, rather than hypoxia itself^11^. The liver has also been identified as one of the most frequently affected extra-cranial organs in infants with HIE^46^ with reported incidence ranging widely from 22% to 80%^11,27,35,53,54^ and in this cohort, it is the first and most injured organ. Prior studies suggest that infants with severe NE are 13 times more likely to have elevated liver enzymes.^39^ In line with the literature, our study demonstrated a high rate of liver dysfunction in both the mild and moderate/severe groups (45% vs. 67% respectively) with association between the severity of NE based on the Sarnat score and liver dysfunction (AST *r* = 0.032, ALT *r* = 0.089 *p* = 0.022).

### Multiple organ Injury

Nearly a third of our cohort had evidence of at least one organ injury, with multiorgan injury ranging from 7% in mild cases to 13% in moderate/severe cases. Hepatic and cardiac injuries were more frequent in the moderate NE group compared to the severe group, likely due to the larger sample size in the moderate group. This increased variability in outcomes may not reflect a true difference in severity-related organ damage. A total of 30 infants died, including three (10%) from the mild NE group. Upon further examination, all three had cardiac involvement, with one also having liver injury, another kidney injury, and the third having injuries to the heart, liver, and kidneys. The heart’s vulnerability to hypoxia and ischemia likely contributed to its involvement in all cases.

MOD and its components (cardiac dysfunction, liver failure, renal impairment) could be contributing to the clinical presentation of mild encephalopathy. Our study, consistent with prior reports, found a significant association between MOD and mortality.^33,55,56^ Even in the mild NE group, we report three infants progressed to more severe disease with disseminated intravascular coagulation, respiratory failure, and death. Neonates with renal, hepatic, and cardiac dysfunction were at higher risk of mortality, highlighting the multifactorial nature of HIE outcomes.

We recognize additionally that the burden of MOD in neonates with moderate to severe NE may be underestimated in our study as those infants received therapeutic hypothermia (TH). TH can mitigate organ injury even in the first day of life, compared to mild NE cases that do not receive TH. Despite this, and important to counseling our cohort demonstrates improved clinical tests by day 3, regardless of TH use or NE severity.

### Strengths and limitations

Study strengths include the large cohort of neonates across the entire NE severity spectrum, universal blood gas screening for acidosis, comprehensive neurological assessment within six hours to stratify encephalopathy, and specific MOD laboratory testing which were obtained as standardized protocols for all infants as part of our NeuroNICU program.

Limitations include the varying definitions of organ-specific injury based on laboratory testing on the first and third day of life. For example, KDIGO kidney scoring is not applicable as it necessitates measurements on day 7 of life.^57^ Additionally, our study lacks comprehensive data on maternal creatinine levels in our dataset, which restricted our ability to perform direct comparisons between neonatal and maternal creatinine values. However, we used similar definitions from published studies on MOD normative data.^32–35,37,41^ We also did not account for potential reclassification of infants across NE severity categories over time, but future studies should account for the dynamic progression of NE severity and the involvement of other organ systems. A key limitation of this study is the lack of healthy controls; we use fetal acidosis and the range of mild encephalopathy for comparisons as well as our laboratory normative references for newborns. While no significant differences were observed between neonates with complete and missing data, unmeasured confounding cannot be ruled out. Future studies with more complete datasets could help confirm these findings.

## Conclusion

While neonates with moderate and severe NE have traditionally been closely monitored for MOD, our study highlights a notable rate of organ injury in neonates with mild NE, emphasizing the importance of MOD in this vulnerable population. This study supports the need for screening and monitoring for MOD in neonates with NE, regardless of encephalopathy severity. However, the observed association between mild NE and MOD does not establish causality, and further prospective studies with robust designs are essential to clarify the directionality and underlying mechanisms of this relationship. By building on these findings, future research can aim to refine early detection and treatment strategies, ultimately mitigating the systemic impact of HIE and improving both short- and long-term outcomes for these neonates.

## Summary

### What’s Known on this subject and what this study adds

- Hypoxic-ischemic insult can cause multi-organ dysfunction (MOD).
- Studies showed organ injuries in neonates with moderate and severe neonatal encephalopathy (NE).
- Lack of research exists on MOD in mild NE.
- Another knowledge gap is whether the extent of MOD matches the severity of NE.

## Data Availability

The data supporting the findings of this study are available upon request from the corresponding author.
